# Layer-dependent morphologies of silver on n-layer graphene

**DOI:** 10.1186/1556-276X-7-618

**Published:** 2012-11-09

**Authors:** Cheng-wen Huang, Hsing-Ying Lin, Chen-Han Huang, Ren-Jye Shiue, Wei-Hua Wang, Chih-Yi Liu, Hsiang-Chen Chui

**Affiliations:** 1 Department of Photonics, National Cheng Kung University, Tainan, 70101, Taiwan; 2 Center for Nano Bio-Detection, National Chung Cheng University, Chiayi, 621, Taiwan; 3 Institute of Atomic and Molecular Sciences, Academia Sinica, Taipei, 10617, Taiwan; 4 Advanced Optoelectronic Technology Center, National Cheng Kung University, Tainan, 70101, Taiwan

**Keywords:** Graphene, Nanoparticle growth mechanisms, Diffusion difference barriers, 68.65.Pq (graphene films), 68.70.+w (whiskers and dendrites), 78.67.Wj (optical properties of graphene)

## Abstract

The distributions of sizes of silver nanoparticles that were deposited on monolayer, bilayer, and trilayer graphene films were observed. Deposition was carried out by thermal evaporation and the graphene films, placed on SiO_2_/Si substrates, were obtained by the mechanical splitting of graphite. Before the deposition, optical microscopy and Raman spectroscopy were utilized to identify the number of the graphene layers. After the deposition, scanning electron microscopy was used to observe the morphologies of the particles. Systematic analysis revealed that the average sizes of the nanoparticles increased with the number of graphene layers. The density of nanoparticles decreased as the number of graphene layers increased, revealing a large variation in the surface diffusion strength of nanoparticles on the different substrates. The mechanisms of formation of these layer-dependent morphologies of silver on n-layer graphene are related to the surface free energy and surface diffusion of the n-layer graphene. The effect of the substrate such as SiO_2_/Si was investigated by fabricating suspended graphene, and the size and density were similar to those of supported graphene. Based on a comparison of the results, the different morphologies of the silver nanoparticles on different graphene layers were theorized to be caused only by the variation of the diffusion barriers with the number of layers of graphene.

## Background

A single atomic layer of graphene is the thinnest sp^2^ allotrope of carbon. It, therefore, has various unique electrical and optical properties of interest to scientists and technologists [1-3]. Graphene samples are widely fabricated by the micromechanical cleavage of highly oriented pyrolytic graphite (HOPG) with scotch tape. Layers of oxides such as SiO_2_ and Al_2_O_3_ with special thickness between graphene and the substrate are typically used to make graphene optically visible [4-7]. The effect of the substrate on Raman measurements has been widely investigated [8]. Raman and surface-enhanced Raman spectroscopy have been widely utilized to elucidate the vibration properties of materials [9-14]. Recently, they have been used as powerful techniques for characterizing the phonons of graphene [15-20]. The profile and peak position of the Raman second-order (2D) band can be used to determine the number of graphene layers [21,22].

In this work, layers of graphene were fabricated in a sample by micromechanical cleavage. The number of layers of graphene was determined by micro-Raman spectroscopy and optical microscopy. After silver nanoparticles were deposited on the sample using a thermal deposition system, the distribution and sizes of the particles on flakes with different numbers of layers were systematically analyzed. To analyze the effect of the substrate, suspended graphene was fabricated, and the size and density thereof were found to be similar to those of supported graphene. The different results for the mono-, bi-, and tri-layer graphene are theorized to be caused only by the variation among the diffusion barriers of the various graphene layers, which provides a method of determining the number of graphene layers and provides information that can be utilized to elucidate the interaction between a graphene flake and its substrate.

## Methods

**Figure 1:**
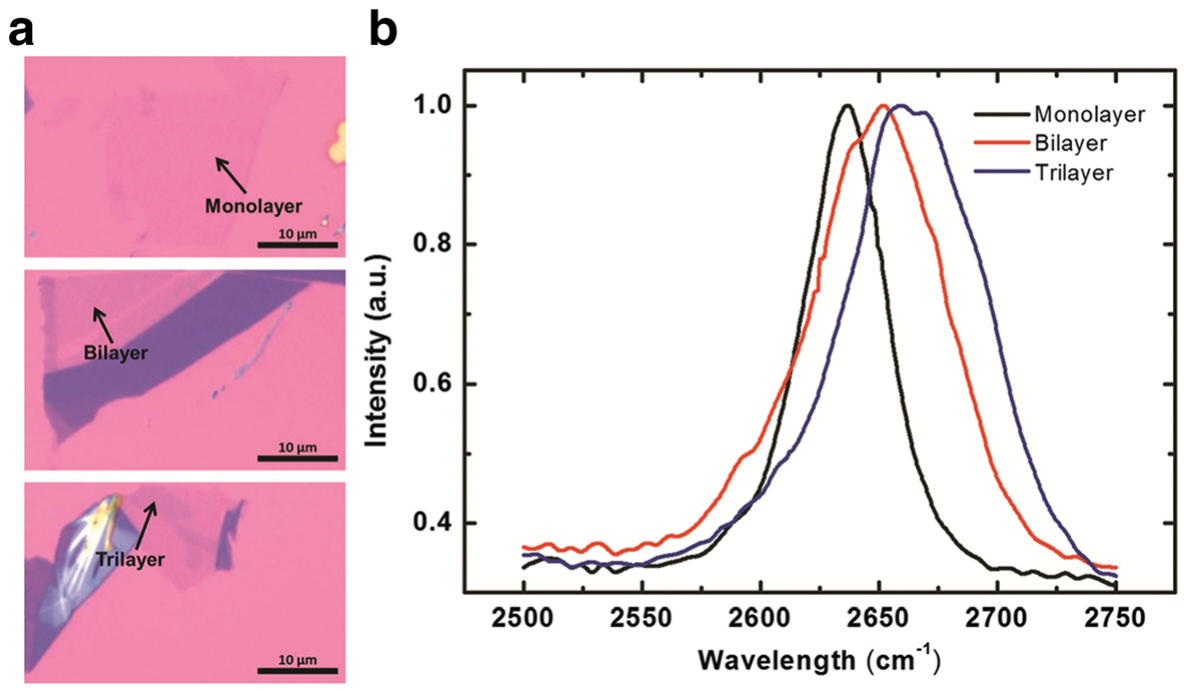
**Graphene with different layers on SiO**_**2**_**/Si substrate.** (**a**) Optical image and (**b**) Raman spectra of graphene with different layers on SiO_2_/Si substrate, which are measured and collected using 632.8-nm laser.

A graphene flake was fabricated by micromechanical cleavage with scotch tape. It was capped with 300-nm-thick layer of SiO_2_ over a Si substrate. Graphene flakes that contained different numbers of graphene layers were distributed on different areas on the same sample. The variation in the color contrast with the number of graphene layers is observed under an optical microscope, as presented in Figure 1a. In the determination of the number of graphene layers, various shapes and 2D bandwidths of graphene were observed by obtaining Raman spectra of different areas, as presented in Figure 1b. A 633-nm He-Ne laser was the excitation light source. The laser beam was focused by a ×50 objective lens (NA = 0.75) on the sample with a focal spot size of approximately 0.5 μm, equal to the spatial resolution of the Raman system. Finally, the radiation was sent to a 55 cm spectrometer and with a liquid-nitrogen-cooled charge-coupled device for spectral recording. According to previous reviews [21,22], peak of the 2D band of graphene became broader and was blue-shifted as the number of graphene layers increased. Based on the results obtained herein, the shape and bandwidth of the monolayer graphene were symmetrical and narrow, respectively, as in the top image in Figure 1a. The middle and bottom images in Figure 1a can be identified as the bilayer and trilayer graphene by the same analysis.

## Results and discussion

**Figure 2:**
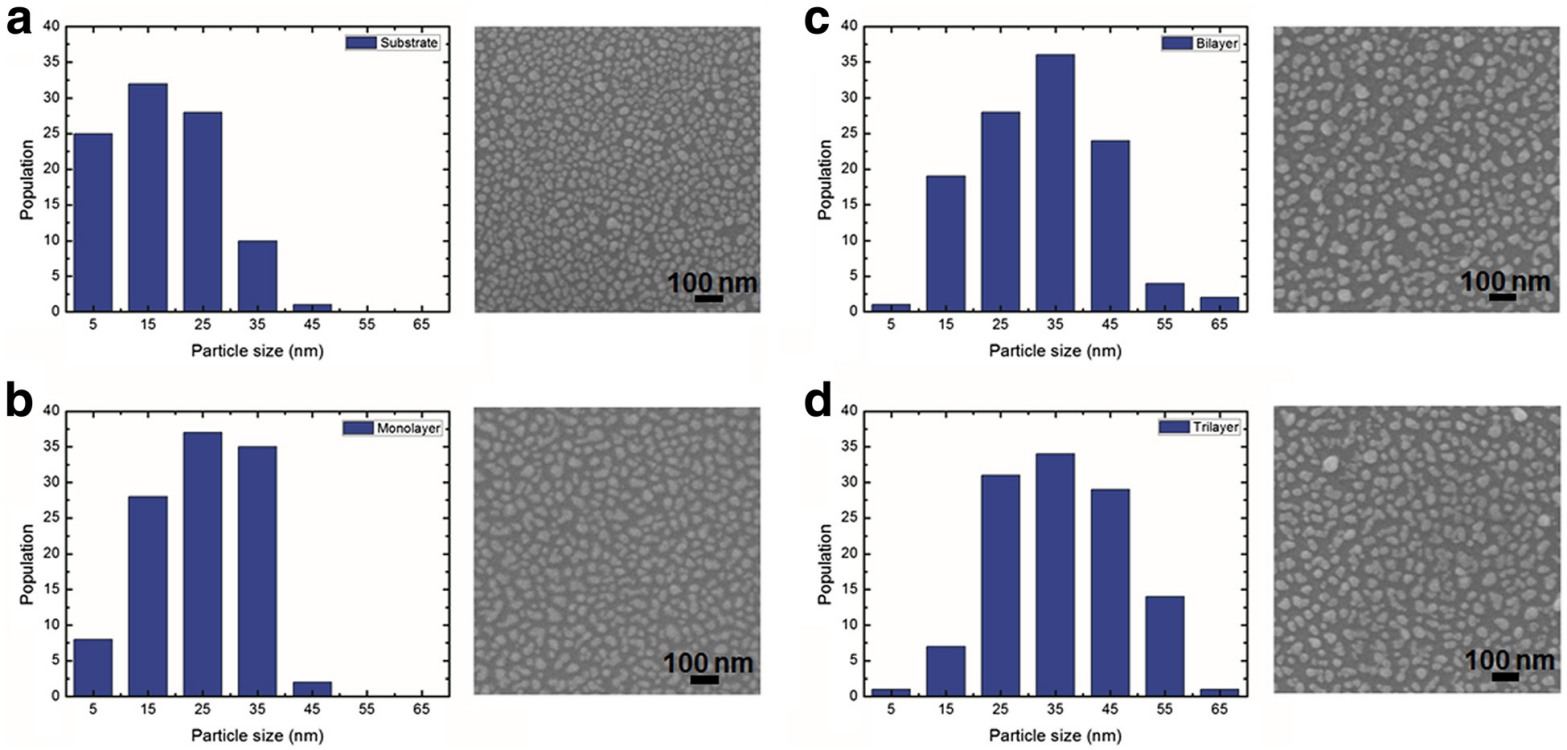
**Histograms and SEM images of silver nanoparticles.** (**a**) Substrate, (**b**) monolayer, (**c**) bilayer, and (**d**) trilayer graphene flakes.

Silver nanoparticles were deposited on graphene flakes at a rate of 0.5 nm/min using a thermal deposition system to a thickness of 5 nm at a fixed temperature of the deposition system of 300 K. To elucidate the surface diffusion, the sample after deposition was maintained at 373 K in a vacuum for 1 h. To study the surfaces of the graphene flakes, scanning electron microscopy (SEM) images of the distribution of nanoparticles on the SiO_2_/Si substrate and the monolayer, bilayer, and trilayer graphene flakes were obtained, and shown on right-hand sides of Figure 2a,b,c,d, respectively. The density of nanoparticles decreased as the number of graphene layers increased and was highest on the SiO_2_/Si substrate.

To investigate the density and size of the nanoparticles, average size and density were determined by histogram analysis. The histograms, on the left-hand sides of Figure 2a,b,c,d, demonstrate the distributions of nanoparticles on the SiO_2_/Si substrate, monolayer, bilayer, and trilayer graphene. The sizes of the nanoparticles on the monolayer graphene flake are distributed in the range of 0 to 50 nm, whereas those of the nanoparticles on the trilayer graphene flake were distributed in the range of 10 to 70 nm. Whereas the sizes of the nanoparticles on the SiO_2_/Si substrate were distributed in the range of 0 to 50 nm, the majority of them were in the range of 10 to 30 nm.

**Figure 3:**
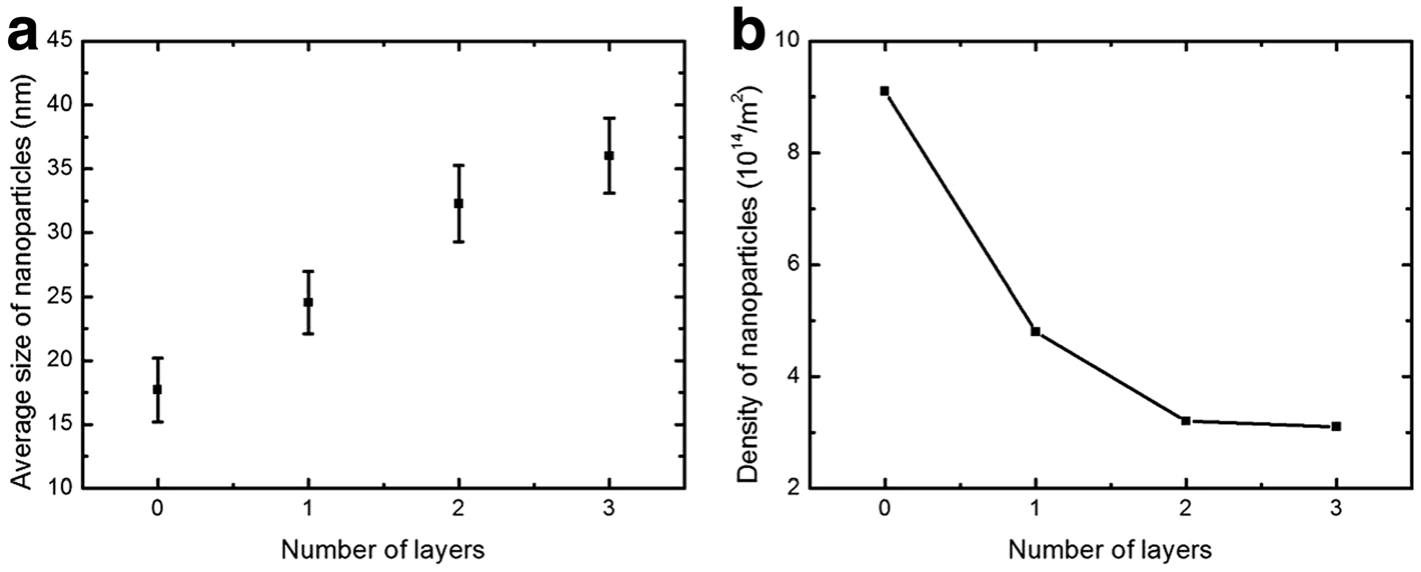
**Nanoparticles on different graphene layers supported by a Si/SiO**_**2**_**substrate.** (**a**) Average size and (**b**) density.

In Figure 3, zero graphene layers represent the SiO_2_/Si substrate without any graphene flake. The nanoparticles on the trilayer graphene are largest, with a mean size of approximately 36 nm; as the number of graphene layers decreased, the average size fell to 32, 25, and 18 nm. The density of nanoparticles on the trilayer graphene was also the lowest, at around 3.1 × 10^14^/m^2^. Reducing the number of graphene layers increased the density from 3.1 × 10^14^ to 9.1 × 10^14^/m^2^. The density of nanoparticles decreased as the number of graphene layers increased, revealing a large variation in the surface diffusion strength of nanoparticles on the different substrates. The mechanisms of formation of these layer-dependent morphologies of silver on n-layer graphene are related to the surface free energy and surface diffusion of the n-layer graphene. Different morphologies of silver on the differently layered graphene are obtained when silver particles are deposited on the graphene surfaces because of the differences of surface free energy. The variation in surface free energy with number of layers is responsible for the variation in the morphologies of silver on the differently layered graphene. The particles are randomly deposited on the graphene surface, and surface diffusion causes randomly arranged particles to combine to form relative large islands. The surface diffusion coefficient *D* ∝ exp(−*E*_*n*_/*kT*) indicates the interaction between particles and the substrate surface, where *E*_*n*_ and *k* denote the diffusion barrier and the Boltzmann constant, respectively. The density of the particles on the substrate (*N*) can be expressed as *N* ∝ (1/*D*)^1/3^ for isotropic surface diffusion. The diffusion barrier specifies the interaction between particles and the substrate surface. The difference between the diffusion barriers of the substrate and monolayer can be calculated as *ΔE* = *E*_0_ − *E*_1_ = 3*kT* ln(*N*_0_/*N*_1_) = 61.7 *mev* by combining the above equations. The difference between the barriers of the substrates with other numbers of layers can be similarly obtained, and they are 39.1 meV (E_1_-E_2_) between the first and second layers and 3.1 meV (E_2_-E_3_) between the second and third. The difference between the diffusion barriers of silver decreased as the number of graphene layers increased. According to one review [23], the area density of gold nanoparticles on SiO_2_/Si substrate and different graphene layers, which the most is trilayer, is in the range of 9 × 10^13^ to 1.25 × 10^13^/m^2^, and the difference between diffusion barriers of different layers is in the range 500 to 291 meV. The area density of the silver nanoparticles herein is approximately ten times this value, indicating that silver nanoparticles can be more easily prevented from moving along the graphene surface, revealing a stronger interaction between silver and graphene than between gold and graphene.

**Figure 4:**
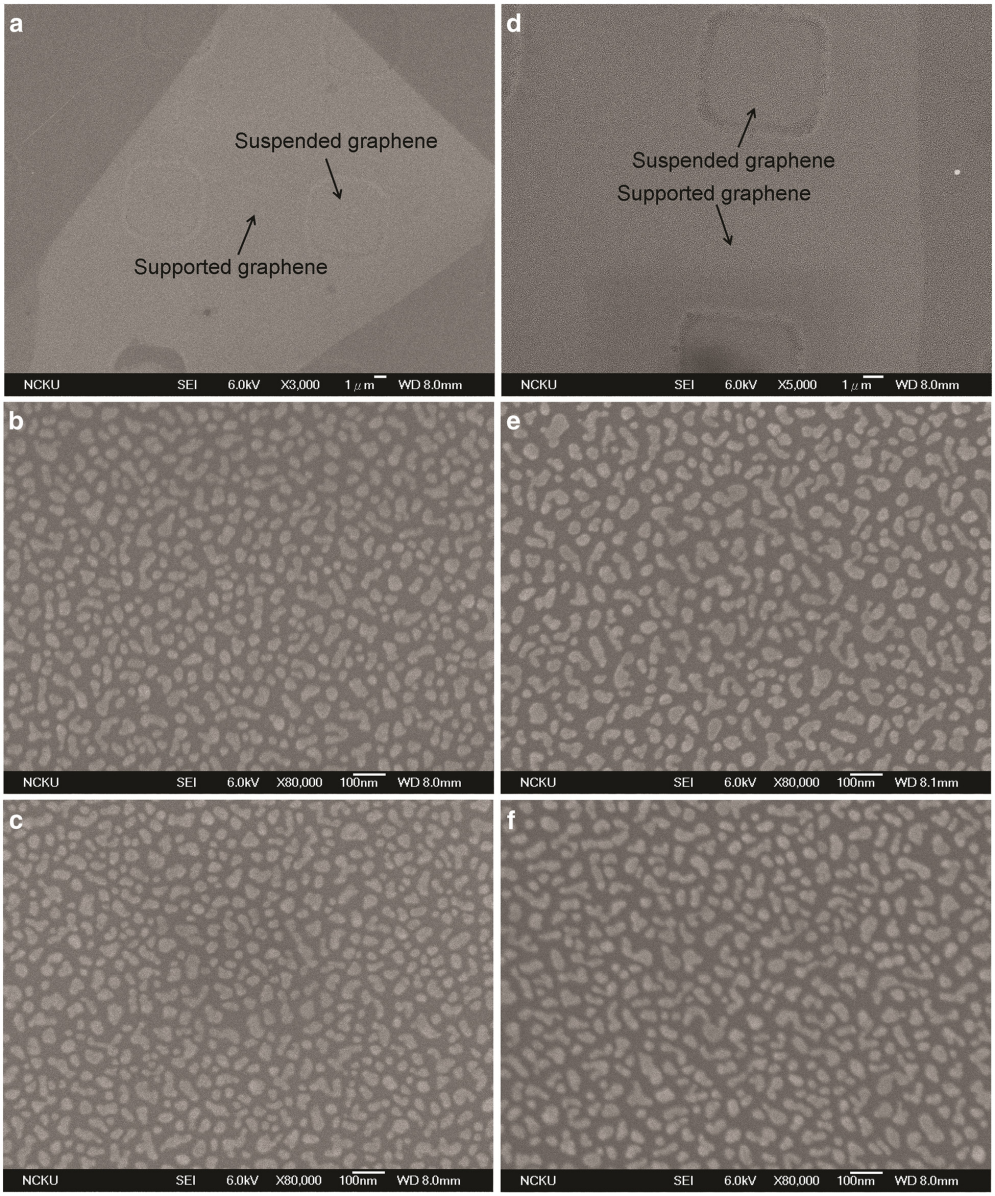
**SEM images of supported and suspended graphene.** Monolayer (left) and bilayer (right).

To determine whether the substrate effect of SiO_2_/Si is responsible for the variation in morphology, suspended graphene was fabricated by mechanical exfoliation of the graphene flakes onto an oxidized silicon wafer. First, ordered squares with areas of 6 μm^2^ were defined by photolithography on an oxidized silicon wafer with an oxide thickness of 300 nm. Reactive ion etching was then used to etch the squares to a depth of 150 nm. Micromechanical cleavage of HOPG with scotch tape was then used to deposit the suspended graphene flakes over the indents. The deposition conditions and silver nanoparticles were the same for both types of graphene. Figure 4 presents the SEM images of the supported and suspended graphenes which were identified as monolayer and bilayer graphene, respectively. Figure 4a presents the monolayer, and Figure 4b,c presents a magnified view of the suspended and supported graphene, respectively. Figure 4d presents the SEM image of the bilayer, and Figure 4e,f presents the SEM images of the suspended and supported graphenes. By the same analysis as above results, the average sizes of the suspended monolayer and bilayer graphenes are calculated as 25 and 34 nm. No clear differences exist between the supported and suspended graphenes, whether they be monolayer or bilayer, suggesting that trilayer graphene will similarly be unaffected. Whether the graphene is supported or suspended, the silver nanoparticles on the bilayer graphene are slightly larger than those of the monolayer, and their area density is lower. Based on the results, the use of a substrate such as SiO_2_ does not affect the distribution of silver nanoparticles on the surface of graphene.

## Conclusions

In this work, the distribution of sizes of silver nanoparticles that were deposited on graphene films with different numbers of atomic layers of graphene was investigated. A systematic analysis revealed that the average size of the nanoparticles increased, and the area density and the difference between diffusion barriers of the nanoparticles decreased as the number of graphene layers increased. To analyze the effect of a substrate such as SiO_2_, suspended graphene was also fabricated. The size and density of suspended graphene were found to be similar to those of the supported graphene. According to these results, only variations in the interactions between n-layer graphene and the silver nanoparticles were responsible for the variation in their distribution.

## Competing interests

The authors declare that they have no competing interests.

## Authors’ contributions

C-WH carried on the experimental parts: the acquisition, analysis, and interpretation of data. C-WH was also involved in drafting the manuscript. H-YL and C-HH took the analysis and interpretation of data. They also had been involved in revising the manuscript. R-JS and W-HW (Institute of Atomic and Molecular Sciences, Academia Sinica) prepared the graphene samples using micromechanical method and obtained the OM images. C-YL has made substantial contributions to the conception and design of the study, and in the critical revision of the manuscript for important intellectual content. H-CC, the corresponding author, had made substantial contributions to conception and design, and had been involved in drafting the manuscript and revising it critically for important intellectual content. All authors read and approved the final manuscript.

## Authors’ information

C-WH received his BS degree in Electrical Engineering from the National University of Kaohsiung, Kaohsiung, Taiwan, in 2008. He studied his MS degree in 2008 and Ph.D. degree directly in 2009. Currently, he is a Ph.D. candidate in the Department of Photonics, National Cheng Kung University, Tainan, Taiwan. He focuses on the property of graphene and surface plasmon resonance of nanoparticles. H-YL received her doctoral degree from the Institute of Biomedical Engineering, National Cheng Kung University in 2010. Her current research interest focuses on the SERS properties of graphene and possible sensing application. C-HH received his doctoral degree from the Institute of Electro-Optical Science and Engineering, National Cheng Kung University in 2009. His current research interest focuses on the Raman and SERS properties of graphene incorporating with metal nanoparticles.
